# Predicting Seagoing Ship Energy Efficiency from the Operational Data

**DOI:** 10.3390/s21082832

**Published:** 2021-04-17

**Authors:** Aleksandar Vorkapić, Radoslav Radonja, Sanda Martinčić-Ipšić

**Affiliations:** 1 Faculty of Maritime Studies, University of Rijeka, 51000 Rijeka, Croatia; radonja@pfri.hr; 2 Department of Informatics and Centre for Artificial Intelligence and Cybersecurity, University of Rijeka, 51000 Rijeka, Croatia; smarti@inf.uniri.hr

**Keywords:** ship operational performance, energy-efficient shipping, data mining, machine learning, linear regression, multilayer perceptron, support vector machine, random forest

## Abstract

This paper presents the application of machine learning (ML) methods in setting up a model with the aim of predicting the energy efficiency of seagoing ships in the case of a vessel for the transport of liquefied petroleum gas (LPG). The ML algorithm is learned from shipboard automation system measurement data, noon logbook reports, and related meteorological and oceanographic data. The model is tested with generalized linear model (GLM) regression, multilayer preceptor (MLP), support vector machine (SVM), and random forest (RF). Upon verification of modeling framework and analyzing the results to improve the prediction accuracy, the best numeric prediction algorithm is selected based on standard evaluation metrics for regression, i.e., primarily root mean square error (RMSE) and relative absolute error (RAE). Experimental results show that, by taking an adequate combination and processing of relevant measurement data, RF exhibits the lowest RMSE of 17.2632 and RAE 2.304%. Furthermore, this paper elaborates the selection of measurement data, the analysis of input parameters, and their significance in building the prediction model and selection of suitable output variables by the ship’s energy efficiency management plan (SEEMP). In addition, discretization was introduced to allow the end user to interpret the prediction results, placing them in the context of the actual ship operations. The results presented in this research can assist in setting up a decision support system whenever energy consumption savings in a marine transport are at stake.

## 1. Introduction

Measurement data from the ship automation system provides more information than the operator can comprehend by reviewing them. To make interpretation easier for operators, the makers of such systems present the data in the form of graphs. For the onboard measurement data to be fully utilized, they need to be opposed to the meteorological and oceanographic conditions on the voyage, and computer-processed to extract a pattern, enable future prediction, and learn the new knowledge. Using data mining and machine learning (ML) methods, the operator can better understand the underlying relationships among the onboard measurement and related meteorological and oceanographic data. The ability to predict operational parameters will help decide on more efficient use of energy sources.

Machine learning algorithms learn the model on a set of input variables, and in general, the quality of learning depends on the selection. Furthermore, the selection of input variables is necessary because the processing time, when using an unlimited number, can be significantly increased in real models. Therefore, this research deals with the selection of input variables with regard to their reduced number and finding the optimum between the quality of predictions and the number of input variables. With large datasets, a detailed look into a black box and data preparation for model learning is required [1]. The influence of input variables on the prediction result is proportional to the importance of individual attributes in model construction. However, the model cannot know with certainty what the quality of prediction would be if some data were removed because in that case, the model would be built differently. Equally, it is not possible to know whether all scenarios of navigational conditions are covered, i.e., whether a sufficient amount of data has been collected and whether the model has been overlearned. Therefore, expert interpretation of the results concerning different combinations of input variables is required.

In the case of ships for the transport of liquefied gas, the amount of fuel required for the liquefaction of cargo is more than 10% of the amount needed for propulsion (on the case LPG vessel, the liquefaction plant takes 1396.1 kW for cargo cooling down in navigation against 11,160 kW required for continuous propulsion service operation, or 12.5%). Therefore, models based on propulsion energy consumption are not accurate enough to be able to address the ship’s energy efficiency successfully.

Predictive modeling is a procedure in which a model is built to predict the value of a selected output variable from known values of input variables, and uses regression and classification algorithms. Since ship operational parameters are regressional, regression algorithms are therefore chosen as a logical choice, while classification is used for reasons of simplified interpretation of the results. Namely, the numeric output value of the ship’s speed is easily interpreted by the operator. At the same time, the specific fuel consumption of the main propulsion machine is easier to interpret if it is placed in the context of the limits imposed by the manufacturer or ship energy efficiency management plan (SEEMP).

This paper presents a model that successfully predicts the relevant operating parameters by the SEEMP expanded with trim optimization and the need for liquefaction of cargo in ships for the transportation of liquefied gas in the operating envelope and in the case of a ship for the transport of liquefied petroleum gas. The ship for the transport of liquefied petroleum gas was selected due to the availability of data and the built-in cargo liquefaction system, making the proposed model applicable to all tanker ships for the transportation of liquefied gases with installed cargo liquefaction systems. Furthermore, given that the type and size of the selected ship is close to the average size of the ships commonly used on oceangoing voyages, results may be applied to any similar oceangoing merchant ship. An adequate ML algorithm using standard evaluation criteria is selected, a detailed look into the black-box is provided, and the significance of the input variables in the actual conditions over a prolonged period is analyzed.

In the previous research, prediction of core operational parameters, primarily added resistance and fuel consumption modeling methods, are broadly classified into three groups: white-box, black-box, and grey-box. The white-box identification technique derives the engine models by resorting to physical laws, while black-box processes measurement data by mining or ML methods. The grey-box combines the two mentioned methods.

Petersen et al. [2] presented an efficiency model of coastal ferry navigation developed using an artificial neural network (ANN) and a Gaussian process (GP) based on measurement data from voyages in a 1 h and 55-min period. The results obtained by artificial neural networks were better than those obtained by the Gaussian process. The relative propulsion power error was 1.65%, while the error in predicting fuel consumption was 1.50%.

Nielsen and Jelsen [3] described a support system in deciding to change the speed of a ship and the direction of navigation in poor weather conditions in real-time, using a prediction model with the assessment of the wave impact. The proposed model estimates the wave spectrum by combining hydrodynamic modeling and statistical processing of retrieved data. The authors used linear spectral analysis in the statistical processing of the normal distribution (Gaussian curve). At the same time, the Monte Carlo simulation (MCS) and the first-order reliability method (FORM) process the data that monitor the discrete functions. The model is based on data collected from eight sensors installed on the deck of the test ship. The measured data were acceleration, wave height, green water sensor data, and data retrieved from the stress sensor. The system gives promising results, but a more considerable amount of data is needed to validate it fully.

In their research, Ruihua Lu et al. [4] set up a model of support in deciding on the choice of the appropriate navigation direction with monitoring of hull and propeller fouling, as well as degradation of the main propulsion engine performance, using a modified method [5] for predicting added resistance. The modification proposed by the authors consists of added five-year noon reports from two ships (Suezmax (Suezmax ships are adapted for the navigation of the Suez Canal in a state of cargo. The term is used almost exclusively for tankers) and Aframax (Aframax is the name for an oil tanker. It is based on the tanker rating system introduced by the Shell Oil Company (Average Freight Rate Assessment, AFRA) to standardize shipping contracts)) and test drive data. Results within an error of 5.12% (Suezmax) and 7.15% (Aframax), respectively, were presented.

Trodden et al. [6] presented a model for planning the fuel consumption and exhaust emissions of tugs and analyzing the initial state, which can then be used to assess the degradation of efficiency during extraction by a software filter algorithm to eliminate all states that do not correspond with the initial. The model processed 43,143 instances of tug speed and fuel consumption measurements over 30 days in different operating modes. The authors showed that the use of eco-speed of the propulsion engine reduced fuel consumption by about 20%, so the presented model was introduced on the tugs where the research was conducted.

Bialystocki [7] presented a computer model for estimating fuel consumption by processing daily (noon) reports of a pure car and truck carrier (PCTC) during 418 days of navigating, with a prediction accuracy in the range of 2.76%.

In his work, Perera [8] dealt with the topic of sensory data processing with an emphasis on the framework of their collection, transmission, and processing. Data were taken from a bulk carrier and processed by clustering, using unsupervised Gaussian mixture models (GMM) with expectation maximization (EM). The corresponding datasets were then displayed graphically, using diagrams. The clustering results are promising, while the collection framework is elaborated in detail.

None of the above papers analyzes the influence, interdependence, and different combinations of the input variables. They are based on reducing the energy consumption of propulsion, which is not sufficient for quality prediction of energy consumption. Previous research does not offer solutions that fit into existing shipboard energy management systems. Hence, in this research we try to traverse this gap. Specifically, this paper introduces the application of machine learning methods on seagoing ship energy efficiency prediction at various navigating speeds in the real oceanographic and weather conditions. Obtained scientific results contribute to better selection and informed analysis of input variables (operational parameters and oceanographic and meteorological data), also regarding their impact on energy efficiency prediction quality. The main contribution of our research proposes the approach for the selection of suitable output variables in compliance with the requirements of the SEEMP and can meet any energy efficiency prediction request scenario. Finally, the novelty in conducted research is also achieved by the expansion of output variables to the need for liquefaction of cargo in ships for the transportation of liquefied gas.

The paper is structured as follows: Section 1 describes the problem and provides previous related work; Section 2 presents data source and data preparation, machine learning algorithms, and evaluation metrics; Section 3 presents the results; Section 4 provides a look into black box and detailed analysis of input and prediction variables, followed by Section 5, which concludes the paper and sets guidelines for future research plans.

## 2. Materials and Methods

Machine learning methods are data-driven, so experimental data and data process analytics standardization is required for model training and verification. In this study, standard open process, the cross-industry standard process for data mining (CRISP-DM) 1.0 is used [9]. The supervised ML methods used in this study are those in which labels for corresponding classes are known in advance. The main goal of supervised data mining is to learn the function *h* for the input set of instances: *Χ → Υ*, where: *X*—set of input variables; *x*—input variable; *Y*—set of output variables; *y*—output variable, so that *h(x)* is a predictor of the corresponding value of 𝑦. 

Among the algorithms used, there are inherent differences according to the format of the output variable; if the output variable *y* is continuous from the set of real numbers y∈*R*, then regression algorithms are used, while the classification is used if the output variable is class and assumes discrete values, for example for the binary case *y* ∈ {*p*, *n*}, where *p* and *n* are discrete class values. Regression refers to numeric prediction, and classification relates to the prediction of labels.

For the training, testing and validation of models, a software toolkit Weka (Weka is an open-access software toolkit developed at the University of Waikato in New Zealand and written in the Java programming language. It is designed to solve data mining tasks using integrated tools for preparation, classification, regression, grouping, association mining, and data visualization) (ver. 3.8.2) is used [10]. In this study we address data mining goals in the following steps (objectives): (1) collection and understanding of data from the shipboard automation system, noon logbook reports, and related meteorological and oceanographic sources; (2) data preparation for machine learning; (3) creation of ML model employing linear regression (GLM), multilayer perceptron (MLP), support vector machines (SVM) (Weka SVM implementation is using sequential minimal optimization for regression, i.e. SMOreg algorithm (invented by John Platt) for solving the quadratic programming problems that arise during the training of support vector machines) and random forest (RF) (the methods are shortly described in Appendix A) capable of predicting the chosen outlet variables based on the input data; (4) selecting the best method based on the standard evaluation metrics by testing the model behavior at a different number of input parameters (variables); and (5) establishing the method for ship operational parameters monitoring. Within the processing model, it is necessary to move back and forth between the individual steps. The outcome of each step determines which phase, or phase task, shall be performed by the next. 

### 2.1. Data Source and Data Preparation

In this study, four groups of data sources from a liquefied petroleum carrier similar to the recent series of the South Korean shipbuilder with a capacity of 54,340 DWT, length 225 m, and width 37 m were used for training and testing, i.e., (1) measurement data from the ship automation system as the primary source, (2) data taken from the electronic chart display and information system (ECDIS), (3) data from noon reports, and (4) available meteorological and oceanographic data in accordance with the geographical position and sailing time. The main engine (ME) is a two-stroke marine diesel engine with one turbocharger unit. The maximum output power is 12,400 kW, considering 15% sea margin and 10% engine margin for fouled ship hull and heavy weather, to satisfy the guaranteed speed of 16.8 knots at the design draft. The engine is a HYUNDAI-MAN B&W 6G60ME-C9.2, while the propeller is HHI Keyless, FPP with four blades, 7400 mm diameter and 5971.06 mm pitch. Data were collected over a total period of 12 months. However, the recordings did not last continuously and measurement data during ship stay in ports, at anchor, or during drifting were not processed. The final recording time of 95 data sources lasted a total of 5042 h. The measurements were performed at a set propulsion engine speed of 89 min^−1^ (normal continuous rating (NCR)), 85 min^−1^ (required speed during charter navigation), and 75 min^−1^ (economic speed above auxiliary blowers switching on pressure) at different loads of the main propulsion engine, resulting in approximately 54 million data points collected from the automation system (Table 1). To ensure repeatability and comparability of measurements, the cooling water temperature of the main propulsion machine is automatically controlled by a temperature controller at 89 °C, while the temperature of the lubricating oil is automatically controlled by a temperature controller between 45 °C and 47 °C.

To minimize errors and eliminate noise and faulty or non-existing signals, the dataset has to be filtered before further analyzing [11]. The problem is that eliminating errors can damage the valuable information, especially when using the deep learning for detecting outliers and extreme values [12,13]. Our approach is to use raw data and rely on measures that are already built into the existing automation and monitoring system architecture; in the event of sensor failure, the system is triggering the alarm. 

Measurement data from all sources have been time-synchronized, normalized, and variables averaged where the time interval was greater.

Given that a number of collected input variables do not affect the target output variables, and some of them are duplicated in different sources (for example, wind speed appearing as sensor data in the group collected from the automation system and from ECDIS, an expert selection was made which resulted in a reduced number of variables from all sources. Furthermore, since variables that are unrelated to the output variable negatively impact the ML performance, they were removed. All data were correlated using Pearson correlation:

(1) $$r=\frac{n(\sum^{}xy)-(\sum^{}x)(\sum^{}y)}{\sqrt{\left[n\sum^{}x^{2}-{\left(\sum^{}x\right)}^{2}\right]\left[n\sum^{}y^{2}-(\sum^{}y^{2})\right]}}\;\;,$$

where *r* is correlation coefficient, *n* is the number of instances, and x and *y* are correlated variables. Variables with correlation above 0.85 were removed. Namely, the performance of linear regression can be reduced if training data has input attributes that are highly correlated. In doing so, we took care not to exclude those variables that are highly correlated in stable navigational and calm weather conditions, but in acceleration or adverse weather come to the fore (main engine specific fuel oil consumption and total fuel oil consumption). After removing highly correlated variables and variables that did not have complete data (even from the alternative sources), 25 variables remained, of which a total of 20 were input and 5 were output or prediction variables. We used 12 variables from main (Table 2) and 13 from secondary data sources (Table 3).

Shaft power (Table 2, variable 2) is measured by the MetaPower‘s torque meter, while temperature (Table 2, variables 8 and 9) and revolutions sensors (Table 2, variable 1) are automation system originally fitted sensors [14]. Fuel oil mass flow (Table 2, variable 2, and 3) is measured by Endress+Hauser’s Proline Promass 80, Coriolis Mass Flow Measuring System made as per ISO-DIN 11,631 with total error of 0.15%. Two flow meters of the same type were installed, one at the engine fuel inlet, the other at the fuel outlet line, and the difference between the readings presents the consumed fuel oil. It is a standard design with a fuel return line for engines that use heavy fuels.

Data from the ship’s noon reports (Table 2, variables 23–25) were used to check the accuracy of the noon reporting against those from other sources, primarily meteorological and oceanographic. To reconcile real-time environments, i.e., real weather conditions in which the vessel was sailing, sea, wave, wind, and current data (Table 2, variables 15–22) were extracted from open-access real-time meteorological and oceanographic databases.

### 2.2. Evaluation Metrics

Standardly, the dataset is split into two independent sets: training and testing (validation) set. The K-fold CV technique partitions the training dataset into k subsets and rotates them k times for the validation, thus expanding the initial quantity of data k times. Usually, k = 10 and each of 10 subsets are systematically applied for training and validation of the models. The set used for training is not used for validating, nor is the validating set used for algorithm training. Final accuracy is an average of each round validation result [10,15]. To test the performance of the trained models in every possible scenario, we employed a 10-fold cross-validation method within the Weka toolkit on 80% of instances on a computer with 1.4 GHz processor and 4 GB 1600 MHz DDR3 memory. Upon selection of the best algorithm, results were confirmed on the remaining 20% of instances reserved for testing. Root mean square error and relative absolute error were primarily used to select the most successful regression algorithm, but additional validation measures were also used because they are easy to interpret. All evaluation measures were standardly implemented in Weka. 

*The correlation coefficient (Cc)* used for regression measures the statistical correlation between the predicted values of ${\hat{y}}_{1},{\hat{y}}_{2},\ldots ,$$\;{\hat{y}}_{n}$ and the true values of $y_{1},y_{2},\ldots ,$$\;y_{n}$. The correlation coefficient ranges from 1, for perfectly correlated results, to 0, when there is no correlation, and −1 when the results are perfectly negatively correlated. The correlation captures slightly different information than other evaluation measures because it depends on the scale in the following sense: if a given set of predictions is taken, the error remains unchanged if all the predictions are multiplied by a constant factor, and the true values remain unchanged. This factor appears in every $S_{\hat{y}y}$ expression in the numerator and in every $\;S_{\hat{y}}$ expression in the denominator, thus invalidating it. The correlation coefficient is calculated according to the following expression:

(2) $$C_{c}=\frac{S_{\hat{y}y}}{\sqrt{S_{\hat{y}}S_{y}}},$$

where:

(3) $$S_{\hat{y}y}=\frac{\sum^{}\left({\hat{y}}_{i}-\bar{\hat{y}}\right)\left(y_{i}-\bar{y}\right)}{n-1},$$

(4) $$\;S_{\hat{y}}=\frac{\sum^{}\left({\hat{y}}_{i}-\bar{\hat{y}}\right)}{n-1},$$

(5) $$\;S_{y}=\frac{\sum^{}\left(y_{i}-\bar{y}\right)}{n-1},$$

and where $\bar{y}$ represents the mean over the predicted values and $\bar{\hat{y}}$ represents the mean over the true values.

*The true positive rate (TPR)* measure is a standard classification measure for evaluating ML algorithms that neglects the classification of negative cases. It reflects the importance of positive cases in the classification of data [16]. It is equal to the ratio of the number of correctly classified cases (true positive, TP) and the total number of positive values (P):

(6) $$TPR=\frac{TP}{P}.$$

The number of total positive values (P) is equal to the sum of correctly classified and false negative values (FN):

(7) P = TP + FN.

To calculate the total TPR for multi-class classification models, Weka outputs a weighted average (WA) which is calculated as the ratio of the sum of all rates of measure per class multiplied by the number of cases according to the classified classes and the total number of specimens:

(8) $$\mathrm{WA}\;=\frac{\sum_{class=1}^{n}\mathrm{measure}\;*\;number\;of\;cases\;per\;class}{number\;of\;instances},$$

where *n* presents number of classes.

*Root mean square error (RMSE)* used for regression and classification is a commonly utilized measure calculated using the expression:

(9) $$RMSE=\sqrt{\frac{{\left({\hat{y}}_{1}-y_{1}\right)}^{2}+\ldots +{\left({\hat{y}}_{n}-y_{n}\right)}^{2}}{n}}.$$

where $\hat{y}$ is the predicted value or class, y is the actual, and n is the sample size.

*Relative absolute error (RAE)* used for regression calculates relative values according to the following expression:

(10) $$RAE=\frac{\left|{\hat{y}}_{1}-y_{1}\right|+\ldots +\left|{\hat{y}}_{n}-y_{n}\right|}{\left|\bar{y}-y_{1}\right|+\ldots +\left|y-y_{n}\right|}\;x100\%.$$

*A confusion matrix* is used to describe the effectiveness of a classifier—when the actual value of the data being estimated is known. The results are often presented in the form of a two-dimensional confusion matrix (Table 4). The positive/negative label refers to the predicted outcome of the experiment, while the truth/false refers to the actual outcome. Positive results correspond to numbers on the main diagonal, and negative results are in non-diagonal cells and have preferably low, ideally zero, values.

*Model building time,* although not a standard validation measure, since all algorithms learn from the same dataset, can be useful in selecting the appropriate algorithm, especially since, in the case of maritime practice, it may indeed be a large amount of data.

## 3. Results

Numeric prediction with 20 input variables and main propulsion engine fuel consumption as an output variable was used to select the most successful ML algorithm. 

The results of evaluating the efficiency of GLM (weka/functions/LinearRegression) (Table 5) confirmed that the effect of the basic regression method is motivating for the transition to more complex numeric prediction algorithms. MLP (weka/functions/MultilayerPerceptron) revealed best results with one hidden layer with 20 nodes, 0.1 momentum, and 0.2 learning rate. Support vector machines (weka/functions/SMOreg), the regression prediction algorithm for the presented results in Table 5, used the PUK. One of the basic difficulties of the ML algorithm is the problem of model overlearning. In RF (weka/trees/RandomForest), this is elegantly solved with enough individual decision trees that use bottom-up pruning techniques and different weights to apply attributes that contain the learning data [17]. In the case of MLP, there is a demanding procedure for finding the optimal parameters for building a successful model. The time required for the construction of RF is much shorter.

After analyzing the prediction performance of the four regression methods, the most successful algorithm was selected based on standard evaluation measures. Experimental results and a comparison of algorithm evaluation results show that GLM achieved the worst results, while MLP, and in particular SVM, and RF achieved comparable regression results (Table 5). Since random forest achieved the best results by all evaluation metrics and excellent prediction results within acceptable learning time, and with the least model learning intervention, it can be concluded that random forests seem to be the best option for shipboard fuel consumption prediction practical application. 

The results of the presented regression methods show that by applying the appropriate algorithm it is possible to predict with high accuracy the fuel consumption of the main propulsion machine. The choice of the method of RF algorithm does not exclude the possibility of applying other successful algorithms in solving specific problems and where it is desirable for the properties that such algorithms possess.

## 4. Discussion

With complex datasets, more than accurate prediction is needed, i.e., quality information on the relationship between input and output variables, with a detailed look in the black box and data preparation for model learning, is required [1].

### 4.1. The Analysis of the Influence of Input Variables on Prediction Accuracy

The random forest algorithm learned the model using selected 20 input variables, and the general rule is that RF does not need to reduce the number of input variables [18]. However, in practice, the selection is nevertheless necessary because the prediction time, when using an unlimited number of input variables, can be significantly increased in real models. Furthermore, the transfer and processing of a smaller amount of data is always an advantage because such systems are generally faster and more flexible. 

Upon finding the most successful combination of input variables and the most successful data mining algorithm, the influence of individual input variables or groups of variables on the output result is additionally analyzed, as well as the evaluation of the performance. 

**Figure 1:**
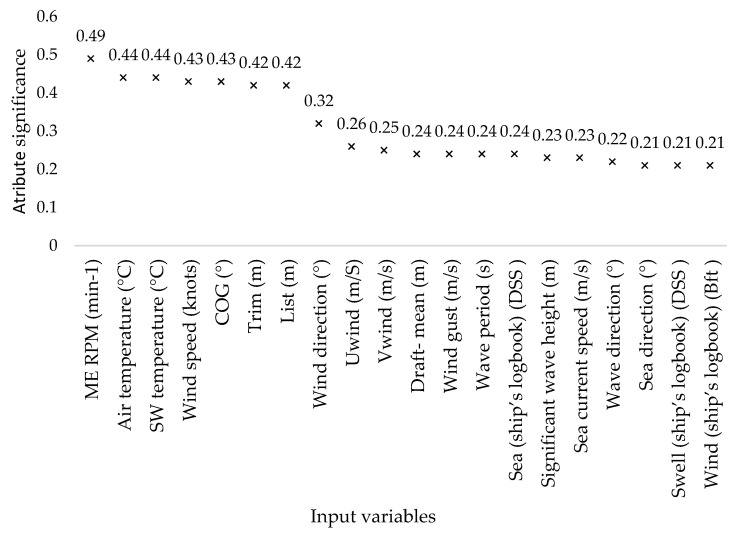
Significance of individual variables (attributes) in the construction of random forest model.

The influence of input variables on the prediction result (Figure 1) is proportional to the importance of individual attributes in building random forest models. However, the out of bag (OOB) method cannot know with certainty how the attributes would behave if some were removed because in that scenario the tree would be built differently. Equally, it is not possible to know whether all scenarios of external navigation conditions are covered, i.e., whether a sufficient amount of data has been collected and whether the model has been overlearned. Therefore, expert interpretation of the results for different combinations of input variables is required. 

Discretization is performed for easier applicability in ship operations where it is important to timely predict the class of fuel consumption as support when making decisions related to the operation of the ship. Outlet variable, i.e., main propulsion engine fuel consumption, is divided into five groups (labels) which correspond to the fuel consumption according to the charter party: class a < 1333, 1333 ≤ b < 1583, 1583 ≤ c < 1770, 1770 ≤ d ≤ 1916, and e > 1916, in kg/h. Classes can be further optimized depending on the need to compare the numeric results, such as a comparison with the fuel consumption from the SEEMP, the values measured during the sea trial, or from the factory test bench. 

The random forest classification algorithm built the model in 5.31 s with 18,018 out of 18,499, or 97.40% of, correctly classified instances and an RMSE of 0.0895. The confusion matrix is shown in the Appendix B as Table A1.

**Figure 2:**
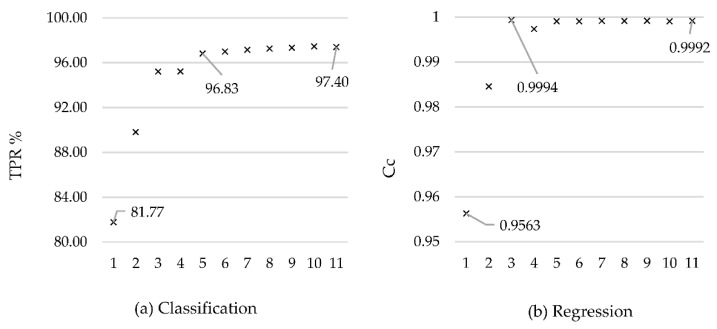
(**a**) Accurately classified instances (true positive rate (TPR)) and (**b**) correlation coefficients (Cc) for regression.

A graphical presentation (Figure 2) of accurately classified instances (TPR) (classification/left) and correlation coefficients (Cc) (regression/right) in combinations of input signals according to the groups (1–11) described in Table 6 gives an overview on how input variable selection affects random forest classification and regression result.

Similar prediction results for classification and regression are to be expected, given that both models are learned on the same data with the same input signals, only the output variable is written differently—once as a classification, and the second time as a numerical value for regression.

The model with only one input variable, i.e., ME rpm (group 1, Table 6) is able to predict fuel consumption with 81.77% classification accuracy or 0.9563 correlation coefficient in regression. Without this variable it is impossible to predict fuel consumption for a ship in navigation, so it remains in the model in all combinations.

Data from the ship’s noon reports (group 2) achieve a weaker result than collected publicly available meteorological and oceanographic data (group 3), contributing more to model construction. By including meteorological and oceanographic data and noon report data, a model was built to predict fuel consumption with an already acceptable accuracy of 95.23% of the classification, and correlation coefficient 0.9974 in regression (group 4). The reason for the weaker results of external influences from ship noon reports may be a larger time interval and a smaller total number of variables, and a human factor that does not read with complete accuracy. Humans are a potentially reliable measuring instrument for measuring a range of quantities. However, it is an entirely different question of how metrically accurate and usable this measurement will be. The influence of wind direction and speed from the ship’s anemometer corrects the previously mentioned external factors and improves the overall prediction. This information can be used in future research as a measure of the assessment of collected publicly available oceanographic and meteorological wind data (group 5). The next input variable is the course over ground (group 6), whose significance is in determining the counter angle of external factors to the ship (the input variable of the actual navigation direction is excluded because such information is impossible to know in the future). Air temperature (group 7) and sea temperature (group 8) are equally important, however their real importance in an operational setup is lower than significance derived from the RF model (Figure 1). The explanation stems from the very nature of the collected data, where the adverse weather was in a high percentage accompanied by air (and sea) temperature drop, and therefore the contribution of air and seawater temperature as the manifestation of the swift change of weather conditions is recognized by RF model as highly important (0.44) (Figure 1). Still, in the context of expert explanation when selecting the input variables and their combinations, the temperature significance is lower (Figure 2). The trim (group 9) contributes to the accuracy of the prediction while the list (group 10), despite the fact that significance derived from the RF model is equal to that of the ship’s trim (0.42) (Figure 1), does not contribute to the quality of prediction in an operational setup (Figure 2). The reason stems from the crew’s effort to maintain the ship transversely aligned, while the trim depends on the condition of the cargo, and at the same time, contributes more to fuel consumption. The draft (group 11) moderately contributes to the prediction despite a relatively high impact of 0.24 from the RF attribute significance analysis (Figure 1). In tankers, especially those for the transport of liquefied gases, the draft does not change continuously but it is rather binary, defined by the state of the cargo, i.e., the ship in ballast or cargo condition. 

The random forest algorithm can successfully learn the model through the number of revolutions of the main propulsion machine and external parameters (group 5) in the case of classification with 96.83% of accurately classified instances, while in regression, by combining the number of revolutions of the main propulsion machine and publicly available meteorological and oceanographic data (group 3), can predict fuel consumption with a 0.9994 correlation coefficient. This result is explained by the fact that at the time of data collection, the ship was in navigation at a constant steaming, and the consumption under calm weather conditions is mostly dependent on the number of revolutions of the main propulsion machine. Other variables contribute to the quality of prediction in adverse weather conditions.

In general, it can be concluded that the influence of individual variable follows the importance of attributes in the construction of random forest model (Figure 1). Still, an expert insight into the study of the interdependence of variables is necessary to set up a model that will successfully predict the operating parameters for all cases of the ship’s operating envelope, and subsequently contribute to better energy efficiency. In the case of the random forest, and where the speed of data prediction is at stake, it is possible to exclude one or more variables based on graphically presented results (Figure 2) and discussion of the influences of different combinations of input variables from the previous text. The results indicate that both regression and classification have satisfactory performance in groups 3 and 5, where we include 9 and 14 input variables, respectively. In the case of the need for high precision and the readiness of the model to external conditions that differ greatly from those processed, and when the construction time of the model is not crucial, it is recommended to use all input variables (Table 6).

### 4.2. The Selection of the Output Variables

The selection of output variables followed factors related to propulsion and hull efficiency through the energy efficiency design index (EEDI), SEEMP (parts 1 and 2) [19,20], and other standard operational procedures that can improve the ship energy efficiency. Main output variables are, therefore, main propulsion engine total and specific fuel consumption, ship apparent slip ratio, speed of the ship (over the ground), the longitudinal inclination of the ship (trim), and the need for liquefaction of cargo, while the remaining variables may be calculated by including the known values. 

*The main propulsion engine fuel oil consumption* (FOC) is selected as the primary output variable because the main propulsion engine is, as always, the largest single energy consumer, and it is possible to achieve the most significant energy savings which meet the SEEMP plans and the criteria of the EEDI. The results of RF prediction are presented in the previous section.

*The ship’s speed over ground* (SOG) is used as an independent variable when calculating the energy efficiency indicators of navigation, due to its operational significance when selecting the most favorable route according to sea and weather conditions. Since the classification does not have too many foundations in practical use, only a random forest regression algorithm was used. The RF algorithm built the model in 8.73 s with a C_c_ of 0.9989, RMSE of 0.1542, and RAE 3.1917%. 

*Ship Energy Efficiency Operational Indicator* (EEOI) is a common energy efficiency indicator calculated within a given time period:

(11) $$EEOI=\frac{M_{CO2}}{M_{c}\;x\;d},$$

where *M_CO2_* is the mass of carbon dioxide (t), *M_c_* is the mass of the cargo carried (t), and *d* is the total distance (nm). 

It follows from the above formula that the EEOI depends on fuel consumption (speed, optimization of ship/engine construction), the quantity of cargo, length of travel, part of the journey carried out in ballast, and time spent at anchor, delays in port, repairs, or other inactive conditions. 

From the above, the EEOI can be calculated as per:

(12) $$EEOI=\frac{24\;x\;Fc\;x\;\mathrm{Cf}}{M_{c}\;x\;24\;x\;v\;},$$

where *F_c_* is the fuel consumption (t/hr), C_f_ is the dimensionless coefficient for converting the consumption into the amount of CO_2_ emitted, *Mc* is the mass of the transported cargo (t), and *v* is the speed of the ship (knot). 

Results of EEOI prediction are the product of the prediction results of the main propulsion engine FOC and the SOG. The fuel consumption of other equipment can be easily predicted because it does not depend on weather conditions and the set speed.

To monitor and measure energy efficiency, various *Energy Performance Indicators (EnPI)* built into the SEEMP are used, which can be simple parameters, or more complex, which are considered to represent the energy efficiency of the ship. EnPI for fuel consumption is usually calculated according to the following expression:

(13) $$EnPI_{FC}=\frac{FC-FC_{B}}{FC_{B}},$$

where *F_C_* is fuel consumption (t) and *FC_B_* is initial or comparative fuel consumption. EnPI represents the difference (%) in fuel consumption according to a predetermined basic consumption. Results of $EnPI_{FC}$ prediction are equal to the results for main propulsion FOC alone.

*The specific fuel oil consumption (SFOC)* of the main propulsion engine is used in the calculation of EEDI and, despite the high correlation with the fuel consumption of the main propulsion engine, provides useful information in confirming the actual fuel consumption of the machine according to load and by the manufacturer’s default values. SFOC is calculated in g/kW as the ratio of fuel consumption to the power delivered to the shaft line and represents the efficiency of converting the chemical energy of the fuel into useful work. The SFOC value indicates not only the efficiency of the engine combustion process but also the fuel economy. The RF regression prediction algorithm built the model in 5.87 s with a C_C_ of 0.9887, RMSE of 12.0096, and 9.4625% RAE. For classification purposes, SFOC is divided into four classes corresponding to the consumption according to the test drive results: a < 161, 161 ≤ b < 175, 175 ≤ c ≤ 189, d > 189. Classes can be optimized depending on the results comparison, such as the comparison with the targeted fuel consumption from the SEEMP, or the values measured on the factory test bench. The classification algorithm of the RF method built the model in 5.55 s with 17,962 out of 18,499, or 97.1% of, correctly classified instances, RMSE of 0.103, and the confusion matrix, as shown in the Appendix B, Table A2. 

**Figure 3:**
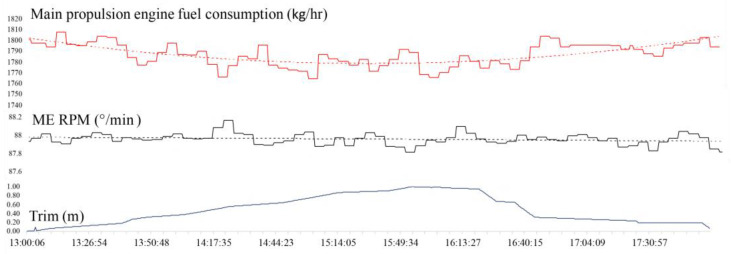
Main propulsion engine fuel oil consumption (FOC) change with change of trim.

*Ship’s slip* is the ratio of the actual speed of the ship and the invested speed of the ship’s propeller. Since the ship’s propeller is immersed in the sea, its actual axial shift is never equal to its pitch, as is the case with a classic screw in a solid material [21]. Therefore, the slip is the ratio between the theoretical and the actual movement, i.e., the propeller pitch and the actual shift, or the loss in propeller pitch. The RF regression prediction built the model in 9.52 s with a C_C_ of 0.995, RMSE of 1.593, and 5.857% RAE. The apparent slip ratio for classification purposes is divided into four classes corresponding to the experimental data: a < 0, 0 ≤ b < 5, 5 ≤ c ≤ 10, d > 10. The RF classification algorithm built the model in 7.34 s with 16,933 out of 18,499, or 91.53% of, correctly classified instances, and RMSE of 0.1856. More information on the results of the classification can be seen in the confusion matrix (Appendix B, Table A3). *Ship’s trim* is used to optimize energy consumption, especially in larger vessels, where the energy savings are more significant (Figure 3). Trim is changed by ballasting or by longitudinally distributing the cargo within the ship cargo holds. In a period of 5 h, the ship’s trim was changed (by ballasting) from the initial 0 m to 1 m of immersion in the bow and back to 0 m. The ship was loaded with 48,394.6 MT of cargo, the weather conditions and the state of the sea did not change, and the control lever was set to 88 rpm and kept stable within 1 rpm. It can be concluded that the subject ship has a lower propulsion fuel consumption when the bow is immersed 1 m more than the stern.

The RF regression prediction algorithm built the model in 7.00 s with a C_C_ of 0.9992, RMSE of 0.0499, and 2.2963% RAE. For classification, trim is divided into five classes corresponding to the limit values from experimental data: a < −1, −1 ≤ b < 0, 0 ≤ c < 1, 1 ≤ d ≤ 2, e > 2. As no measurements were recorded over the −1 m group (ship inclined longitudinally towards the bow over 1 m), the group was omitted from the classification evaluation table. The RF classification algorithm built the model in 4.09 s with 18,116 out of 18,499, or 97.93% of, correctly classified instances, and an RMSE of 0.0879. More information on the results of the classification can be seen in the confusion matrix (Appendix B, Table A4). The set ML model is capable of learning the trim on known data and adequately predicting trim that needs to be maintained in order to match the predicted consumption.

*The need for liquefaction of cargo* on the case of a ship for the transport of liquefied petroleum gas is represented by the total electrical load of the main switchboard. The effective power of electricity required to liquefy cargo is higher than any other operation on a liquefied petroleum gas carrier; hence it is used to monitor the cargo liquefaction. For classification purposes, electrical power is divided into three classes corresponding to the limit values from experimental data and ship’s electrical load analyses drawing: a < 700, 700 ≤ b ≤ 900, d > 900. All three values represent the consumption in navigation so that the first represents the electrical load with reduced consumption, the second normal consumption, and the third consumption with the operation of the cargo liquefaction plant. RF classification algorithm built the model in 5.34 s with 18,145 out of 18,499, or 98.09% of, correctly classified cases, and an RMSE of 0.103. More information on the results of the classification can be seen in the confusion matrix (Appendix B, Table A5). To be able to use liquefaction forecasting as support when planning liquefaction in the future, it is a prerequisite that the decision on liquefaction in the data used for learning is correct. Future research should introduce cargo-related data to make predictions more accurate.

## 5. Conclusions

Models based on data processing are being increasingly used in maritime practices to extract information that can help the operator to operate the ship with better quality and gain additional knowledge. Those models are especially useful with larger amounts of available data being monitored through a longer period. ML methods are being increasingly used to utilize large amounts of data for the purposes of reducing energy consumption.

In this paper, the model is learned from three data groups: automation system operational data, noon reports, and open access real-time meteorological and oceanographic data, and tested with linear regression (GLM), multilayer preceptor (MLP), support vectors machines (SVM), and random forest (RF). By correlation analysis and expert insight evaluation, the number of input variables used in the model was set to 20. Upon verification of modeling framework and analyzing the results to improve the prediction accuracy, the best numeric prediction algorithm was selected based on standard regression evaluation metrics, i.e., root mean square error (RMSE), relative absolute error (RAE), correlation coefficient, and model building time. Experimental results show that, by taking an adequate combination and processing of relevant measurement data, random forests exhibited the lowest RMSE of 17.2632, RAE 2.304%, 0.9992 correlation coefficient, and building time of 6.03 s. Then, the analysis of input variables and their mutual relationship and influence on the quality of prediction was performed. 

The selection of output variables was then approached, considering SEEMP and parameters that could affect the significant reduction of energy consumption on the ship in question. Subsequently, a classification (wherever suitable) was introduced so that the results could be interpreted in terms of shipboard energy efficiency planning, execution, and evaluation. The random forest model obtained results according to the output variables as follows: main propulsion engine fuel consumption, regression results are stated above, and classification TPR 97.9% and RMSE 0.895; ship speed over ground regression results C_c_ of 0.9989, RMSE of 0.1542, and RAE 3.1917%; main propulsion engine specific fuel oil consumption regression C_C_ of 0.9887, RMSE of 12.0096, and RAE 9.4625%, and classification TPR 97.1% and RMSE of 0.103; ship’s slip regression C_C_ of 0.995, RMSE of 1.593, and 5.857% RAE, and classification TPR 91.53% and RMSE of 0.1856; trim C_C_ of 0.9992, RMSE of 0.0499, and 2.2963% RAE and classification TPR 97.93% and RMSE of 0.0879; need for reliquefication classification TPR 98.09% and RMSE of 0.103. It should be noted that the classification results are not fixed and vary depending on the number and type of classes.

A major strength of this study is that all the datasets are from real ship operations and environment, and that it provides a detailed analysis of input and prediction variables. All models are examples of the potential use in describing and predicting shipboard processes and operational parameters. The choice of the appropriate ML algorithm depends on the nature and complexity of the problem, how the data is generated, and the algorithm expected results. 

By selecting the appropriate machine learning algorithm and continuous monitoring and processing of operational data, it is expected to contribute to the adaptation of existing scientific methods of predicting relevant operational parameters in real environmental conditions and encourage further scientific research in improving the energy efficiency of the ship and reducing carbon dioxide footprint. The results of this research can be used in future research related to intelligent data analysis and simulation of ship operation. The proposed model can be used to monitor, predict, and plan more environmentally friendly voyages, as well as to promote safer navigation based on weather routing. The extension to more analytical comparison with other models (i.e., traditional physics-based) remains an open challenge for our future research. 

## Figures and Tables

**Table 1:** Series of data download and recording.

No.	rpm (°/min)	Recording Start		Recording End	
		Latitude	Longitude	Latitude	Longitude
1	87	9°30′59.19′′	311°30′27.36′′	−2°15′47′′	72°7′4.76′′
2	82	19°21′15.06′′	252°59′6.36′′	29°26′3.25′′	264°56′7.43′′
3	86	29°26′3.25′′	264°56′3.45′′	23°21′27.48′′	180°40′20.4′′
4	79	23°36′35.64′′	179°48′55.26′′	35°9′43.68′′	129°28′12′′
5	77	34°30′24.24′′	129°3′44.46′′	23°35′16.14′′	179°50′17.58′′
6	73	23°24′39′′	180°33′23.88′′	12°1′51.9′′	237°55′41.1′′
7	86	21°54′55.5′′	113°12′43.02′′	−4°49′34.44′′	325°16′18.24′′

**Table 2:** List of sensors/tags from the ship automation system.

No	Variable/Tag	Information/Unit
1	Pickup11	ME revolutions per minute (min^−1^)
2	SFOC *	Specific fuel consumption (g/kWh)
3	ME_tot_FL *	ME total FO consumption (mT)
4	Nav_02 *	Ship’s speed over ground (knots)
5	Nav_04	Wind speed from anemometer (knots)
6	Prop_slip *	Apparent slip ratio (%)
7	MSB0-TOT-LOAD *	Total load on busbars (kW)
8	MS114	Ambient air temperature (°C)
9	MW014	Sea water temperature (°C)
10	Trim in meters *	− fore, + aft (m)
11	List in degrees	(°)
12	Draft—mean	(m)

* Used as output/predicted variables.

**Table 3:** List of sensors/tags from secondary data sources.

No	Variable/Tag	Information/Unit
13	ECDIS COG	course over ground (deg)
14	ECDIS wind direction	(deg)
15	Uwind	(m/s)
16	Vwind	(m/s)
17	Wind gust	(m/s)
18	Significant wave height	(m)
19	Wave direction	(°)
20	Wave period	(s)
21	Sea direction	(°)
22	Sea current speed	(m/s)
23	Sea	Douglas sea scale (DSS) (ship’s logbook) (the Douglas sea scale is a measure of the height of the waves and the state of the swell. The scale is expressed from 0 to 9)).
24	Swell	Douglas sea scale (ship’s logbook) (DSS)
25	Wind	Beaufort scale (Bft) (ship’s logbook) (the Beaufort scale is used to evaluate wind strength in the scale from 0 to 12)).

**Table 4:** Confusion matrix.

Outcome	Actual (True/False)	
Predicted (positive/negative)	True positive (TP)	False positive (FP)
	False negative (FN)	True negative (TN)

**Table 5:** Results of application of regression algorithms.

Regression Method	Correlation Coefficient (Cc)	Root Mean Square Error (RMSE)	Relative Absolute Error (RAE) (%)	Model Building Time (s)
Linear regression (GLM)	0.8762	205.5833	36.8504	0.46
Multilayer perceptron (MLP)	0.9907	58.3507	7.0284	120.53
Support vector machines (SVM)	0.963	115.3915	2.2637	2012.82
Random forest (RF)	0.9992	17.2632	2.304	6.03

**Table 6:** List of input variables with combinations in which they enter the prediction model.

No.	Input Variable	Unit	Groups										
			1	2	3	4	5	6	7	8	9	10	11
1	ME rpm	°/min	x	x	x	x	x	x	x	x	x	x	x
5	Wind speed	m/s					x	x	x	x	x	x	x
8	Air temperature	°C							x	x	x	x	x
9	SW temperature	°C								x	x	x	x
10	Trim	m									x	x	x
11	List	°										x	x
12	Draft—mean	m											x
13	COG	°						x	x	x	x	x	x
14	Wind direction	°					x	x	x	x	x	x	x
15	Uwind	m/s			x	x	x	x	x	x	x	x	x
16	Vwind	m/s			x	x	x	x	x	x	x	x	x
17	Wind gust	m/s			x	x	x	x	x	x	x	x	x
18	Significant wave height	m			x	x	x	x	x	x	x	x	x
19	Wave direction	°			x	x	x	x	x	x	x	x	x
20	Wave period	s			x	x	x	x	x	x	x	x	x
21	Sea direction	°			x	x	x	x	x	x	x	x	x
22	Sea current speed	m/s			x	x	x	x	x	x	x	x	x
23	Sea (ship’s logbook)	DSS		x		x	x	x	x	x	x	x	x
24	Swell (ship’s logbook)	DSS		x		x	x	x	x	x	x	x	x
25	Wind (ship’s logbook) (Bft)	Bft		x		x	x	x	x	x	x	x	x
Total number of variables entering the prediction model			1	4	9	12	14	15	16	17	18	19	20

## Data Availability

Data can be found at http://langnet.uniri.hr/resources.html (accessed on 17 March 2021) and was used under the CC-BY-NC-Sa 4.0 license https://creativecommons.org/licenses/by-nc-sa/4.0/ (accessed on 17 March 2021).

## Appendix A

### Overview of Used Machine Learning Methods

The following are basic explanations of the used ML algorithms with highlights on the distinguishing features from standard implementations.

*Linear regression or generalized linear method (GLM)* is a statistical method that computes the coefficients or “weights” of linear expression, and the predicted (“class”) value is the sum of each attribute value multiplied by its weight. If the data lacks a linear dependence, then the process will find the best straight line (linear direction) so that the root mean square error (RMSE) is interpreted as the best result. In Weka, the linear regression algorithm uses the Akaike criterion for selecting the best method. The Akaike information criterion (AIC) estimates the relative quality of statistical method for a given dataset. Given the set of methods for the data, the AIC evaluates the quality of each method relative to every other method. Accordingly, AIC provides the means to select a method, and it is calculated by the expression:

(A1) $$AIC_{result}=-LL+N,$$

where *N* is the number of parameters and LL is the logarithm of the probability with a negative sign to minimize the result [10].

*Multilayer perceptron (MLP)* is a supervised feedforward neural network that simulates the structure of the human brain using a network of artificial neurons with at least three layers of neurons [22]. Each neuron, except the one in the first layer, has a non-linear activation function. The first layer is an input layer, and it contains as many neurons as there are features (input variables) in the training data. The layers are fully connected, which means that each neuron in one layer is connected to each neuron in the next layer. During the regression in a neural network, the input signals travel through neural connections, multiplying with weights before entering the next neuron, where all the values are summed up and added to bias. The calculated value is then passed to the activation function. For the training of the neural network, a backpropagation algorithm is used, which, taking into account the loss function gradient, adjusts weights factors in order to obtain better predictions. Used function for the hidden layers is the sigmoid function [23,24].

*Support vector machines (SVM)* are elegant tools for solving pattern recognition and regression problems. Considering a dataset {(x_1_, y_1_),. . .,(x_n_, y_n_)} with x ∈ Rd (d-dimensional input space) and y ∈ R, support vector tries to find the function f(x), which relates the measured input object to the desired output property of an object [25,24]. The parameters are learned using modifications to the Smola and Schölkopf’s SMO algorithm [26], which demands that the kernel matrix is computed and stored in memory. This requires large memory and involves expensive matrix operations such as Cholesky decomposition of a large submatrix of the kernel matrix [27]. Third, the coding of these algorithms is difficult. Therefore, using modifications to the SMO introduced by Shevade et al. significantly speeds up the SMO algorithm in most of the situations [28].

The Pearson VII function (PUK—Pearson universal kernel) was developed by Karl Pearson in 1895 and it is used as a vector support function. The basic form of the Pearson VII function for curve fitting purposes is calculated by the expression [29]:

(A2) $$f\left(x\right)=\frac{H}{{\left[1+{\left(\frac{2\left(x-x_{0}\right)\sqrt{2^{\frac{1}{\omega }}-1}}{\sigma }\right)}^{2}\right]}^{\omega }}\;,$$

where *H* is the peak height at the center *x_0_* of the peak, and *x* represents the independent variable. The parameters σ and ω control the half-width (also named Pearson width) and the tailing factor of the peak. The main reason to use the Pearson VII function for curve fitting is its flexibility to change, by varying the parameter ω, from a Gaussian shape (ω approximates infinity). Compared to the commonly applied kernel functions, the use of the PUK has two main advantages: on the one hand, it does not require making a selection out of the kernel functions, which simplifies the model building process and saves computing time, and on the other hand, it has a stronger mapping power through which it can properly deal with a large variety of mapping problems.

*Random forest (RF)* is an algorithm used for constructing a forest of random trees by using bagging sampling techniques [30,31]. The trees in random forests are constructed in parallel and there is no interaction between the trees during the building process. RF employed for regression learning operates by constructing a set of decision trees at training time and outputting the class that is the mode of mean prediction of the individual trees. A random forest combines the results of multiple predictions which aggregates prediction of individual decision trees with the modification that prevents trees from being highly correlated. The RF algorithm ensures that the ensemble model does not rely too heavily on any individual feature, and makes fair use of all potentially predictive features. Each tree draws a random sample from the original set of instances when generating its splits, adding a further element of randomness that prevents overfitting.

## Appendix B

**Table A1:** The confusion matrix for main propulsion engine fuel oil consumption set as outlet variable.

Classified as	a	b	c	d	e
a = b	3225	99	23	1	1
b = c	104	6142	6	85	1
c = a	12	3	6398	0	0
d = d	0	120	4	2224	3
e = e	2	1	2	14	29

**Table A2:** The confusion matrix for main propulsion engine specific fuel oil consumption set as outlet variable.

Classified as	a	b	c	d
a = c	13,611	103	20	7
b = b	130	1968	5	107
c = d	29	7	310	15
d = a	6	91	17	2073

**Table A3:** The confusion matrix for apparent slip set as outlet variable.

Classified as	a	b	c	d
a = b	6548	192	293	9
b = a	286	1868	20	21
c = c	288	29	5708	151
d = d	11	22	244	2809

**Table A4:** The confusion matrix for trim set as outlet variable.

Classified as	a	b	c	d
a = e	10,583	107	0	0
b = d	73	1696	0	92
c = b	0	0	207	20
d = c	0	76	15	5630

**Table A5:** The confusion matrix for reliquefication need as outlet variable.

Classified as	a	b	c
a = a	6503	17	38
b = b	80	600	118
c = c	26	75	11,042
